# Role of Anteromedial Cortical Support for Unstable Intertrochanteric Fractures Being Treated With Cephalomedullary Nails

**DOI:** 10.7759/cureus.58303

**Published:** 2024-04-15

**Authors:** Muhammad Asif Rasheed, Muhammad Suhail Amin, Muhammad Nadeem Chaudhry, Faisal Nadeem, Ahmed Mushtaq Khan, Areej Fatima, Irbah Noor

**Affiliations:** 1 Department of Orthopaedics and Traumatology, Combined Military Hospital, Rawalpindi, PAK; 2 Department of Orthopaedics, Army Medical College, Rawalpindi, Rawalpindi, PAK

**Keywords:** secondary stability, pertrochanteric fracture, fracture reduction, cortical support reduction, cephalomedullary nail

## Abstract

Introduction: Reduction quality is of paramount importance for an optimal outcome in unstable pertrochanteric fractures. The non-anatomical functional anteromedial buttress is proposed to prevent impending mechanical complications. We aimed to evaluate the role of anteromedial cortical support in preventing mechanical complications following fixation with the cephalomedullary nail.

Materials and methods: A prospective, single-arm interventional study was conducted in the Orthopaedics Department of a Combined Military Hospital (CMH) in Rawalpindi. The duration of the study was 24 months. Patients were recruited by the purposive sampling technique as per inclusion/exclusion criteria. Preoperatively, the reduction was categorized as per Baumgartner’s and Chang’s criteria. Post-operatively, weight bearing as tolerated was advised. Radiographs prior to discharge for loss of reduction were evaluated. Follow-up radiographic measurements of neck length, neck shaft angle, and their loss as per protocol were done at three and six months.

Results: A total of 202 patients were operated on from October 21 until August 23. Mortality at six months in 39 patients (19.3%) and loss to follow-up in 31 patients (15.3%) resulted in 132 patients with complete follow-up and having developed complications in 12 patients (9.09%). The mean age was 76.3 ± 7.98 years; males were 105 (79.5%), and females were 27 (20.5%). Closed reduction was 58 (43.9%), and additional manoeuvres were required in 74 (56.1%). The mean tip apex distance (TAD) was 24.56 ± 2.76, and the Calcar gap was 5.16 ± 1.27. Cleveland zone centre-centre in 54 (40.9%), inferior-centre in 65 (49.2%), and inferior-posterior (9.9%) were statistically significant for mechanical complications (p≤0.001). There was a significant association between the grading of Chang’s and Baumgartner’s poor groups for the development of mechanical complications (p≤0.001). The mean time to full weight bearing without support was 21 ± 1.22 weeks. The mean Hip Harris score at six months was 69.27 ± 7.68.

Conclusion: Results suggest that anteromedial cortical support can lead to fewer potential mechanical complications at six months. A higher Chang’s grade drives surgeons to engage in additional manoeuvres. Anteromedial cortical support is worth consideration for unstable pertrochanteric fractures.

## Introduction

Geriatric hip fracture is a significant healthcare problem in the modern age. As per a survey, more than 50% of hip fractures will occur in Asia by 2050 due to longer life expectancy and an increase in the older population [1]. This significant increase in the number of patients to be treated in orthopaedic care facilities is a matter of rising concern for the healthcare system in terms of the rise in healthcare expenditure [2]. Osteoporosis is one of the leading underlying causes of these low-energy fractures, with a reported male-to-female ratio of 1:4 [3]. The treatment, being primarily surgical, aims at providing early mobility off the bed and restoring function to avoid medical as well as surgical complications in elderly patients. Traditionally, we have classified geriatric intertrochanteric fractures to distinguish between stable and unstable fractures. Stable fractures are simple fractures in which less communited shaft fragments can be buttressed against the head-neck fragment, leading to minimal impaction upon weight-bearing post-fixation [4]. Unstable fractures, however, can be due to communition or reverse obliquity, which brings translational forces into action responsible for post-operative construct collapse. Current evolving evidence has elaborated the morphological characteristics and fracture line mapping of these intertrochanteric fractures with the help of 3D-computed tomography [5]. It has helped surgeons gain better insight into the fracture pattern, pre-operative planning, and execution of surgical techniques. What has been brought into significant limelight is the always communited posteromedial wall and lesser trochanter, the inability to cherry-pick posterior wall fragments for buttressing, and the lack of role of the posteromedial wall in providing stability to constructs for extracapsular unstable fractures. Intertrochanteric hip fractures (AO/OTA type 31A) are classified by AO into a more meaningful and prognostically significant nomenclature. The comprehension of lateral wall competence and its consideration as a lateral buttress was elaborated by Hsu et al. [6], who defined criteria for ascertaining lateral wall thickness. This criterion was accepted and included in the 2018 version of the AO/OTA Classification Compendium [7]. 

For the stability of constructs, five confounding factors were explained in the literature by Kaufer in 1980 [8]. Two being non-modifiable are quality of bone and fragment geometry, while three that surgeons can influence are fracture reduction quality, implant selection, and implant placement in the Cleveland zone. As of today’s understanding, there is no debate in acceptance of the fact that intramedullary nails are the implant of choice for the fixation of unstable pertrochanteric fractures. The most significant determining factor for construct stability and the avoidance of mechanical complications is the quality of reduction [9,10]. As a principle, the head-neck fragment slides along the axis of the helical blade or screw and comes into contact with the shaft fragment. This secondary stability is required for the construct to prevail upon initial weight-bearing protocols. The incompetence of the lateral wall, as per the current understanding of unstable fractures, leads to construct collapse, which mandates cortex-to-cortex support for secondary stability. The anteromedial wall, the only consistent and uncommunited wall in pertrochanteric fractures, is the only cortex that can act as a buttress to prevent this uncontrolled telescoping and collapse. This non-anatomic reduction sounds new but is actually the reverse of what Gotfried explained for subcapital fractures [11]. Anteromedial buttress reduction is a non-anatomic functional buttress reduction that ensures controlled impaction and telescoping of the head-neck fragment along the axis of the blade of a fixed-angle device, thus providing a mechanically stable environment for the fracture to heal and preventing the collapse of constructs. Chang et al. explained this in 2015 [12], and the author has studied and explored it in various subsequent publications [13].

The rationale of this study is to evaluate the risk of mechanical complications in unstable pertrochanteric fractures being operated on with cephalomedullary nails following an anteromedial cortical support reduction technique. We aim to publish our experience with reduction technique, consideration of anteromedial cortical support, and its utility by orthopaedic surgeons for unstable per-trochanteric fractures. The primary outcome measure chosen is a gross mechanical complication or impending collapse measured by neck shaft angle loss, as recommended in recent literature [14]. To the best of our knowledge, no study so far in this part of the world has been conducted to evaluate the outcome of anteromedial cortical support for unstable pertrochanteric fractures.

## Materials and methods

This prospective, interventional single-arm study (Quasi) was carried out in the Orthopaedics Department, Combined Military Hospital (CMH), Rawalpindi, Pakistan. The duration of the study was two years after approval of the synopsis or six months after follow-up with the last recruited patient. The hospital is the apex tertiary care teaching hospital of the military and serves as a peer referral from the military as well as civilian colleagues for complex trauma cases. Hospital ethical review board approval was sought from the committee via approval letter no. 1416/10/21 dated November 23, 2021.

The sample size was calculated using the WHO sample size calculator. Anticipating that 22.8% of patients with extra-capsular neck femur fractures being operated on would have sustained mechanical complications [15] (with a confidence level of 80% and absolute precision of 0.05), it was necessary to include a sample size of 115 patients in the study.

Patients were recruited using the purposive sampling technique. Patients meeting the following inclusion criteria were included: (i) all patients with a diagnosis of hip fracture extracapsular; (ii) AO OTA classifications 31A2.2 and 31A2.3; (iii) age > 60 years; (iv) traumatic low energy geriatric fracture; (v) ambulatory patient prior to fracture and amenable to surgery. Exclusions categorically included fractures of high energy, patients with polytrauma, patients with neurological complications of fractures, and pathological fractures. Inadequate clinical or radiological follow-up patients were also excluded from the study group.

In the period from October 2021 to August 2023, we operated on 202 patients with unstable intertrochanteric fractures; all patients were adequately optimized for surgery by stratification and preparation by an anesthetist and physician. Patients were planned for surgery with proximal femoral nail antirotation (7S Medical, PERICLES II Proximal Femoral Nail, 125 degrees, Spiral Blade, 4.9 mm locking screw torx, in static mode). Informed consent was obtained for all patients enrolled in this study. Patients' age, sex, contact information, comorbidities, fracture type, and classification as per AO/OTA and Boyde Griffin were endorsed on the research proforma.

All patients were operated on a traction table with the standard position of the Flourscopic C arm for intraoperative assessment of fracture. Closed reduction was achieved by appropriate traction, adduction, and foot position being internally rotated. The patient was prepared and draped, and anteroposterior (AP) and lateral (Lat) views were assessed for the desired reduction position. Additional manoeuvres, if needed, were endorsed, i.e., manual push, Hohmann spike, Schanz pin, Cobb’s elevator, bone hook, or open reduction.

**Figure 1:**
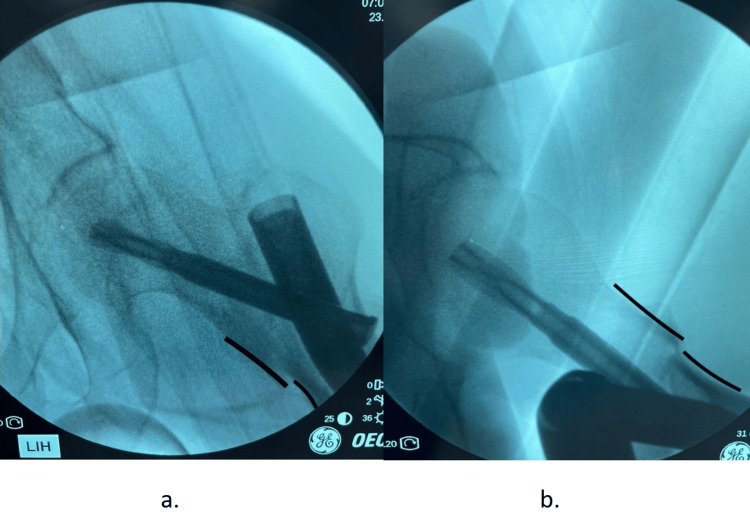
Positive antero-medial cortical support in anteroposterior (a) and lateral (b) per-operative views of image intensifier (a) Anteroposterior view "*Positive"*: the head-neck fragment is supero-medial to shaft fragment; (b) lateral view *"Positive"*:* *the anterior cortex head-neck fragment is lying one cortical thickness above the shaft fragment.

In all cases, a greater trochanter entry point was made, and long nails are a routine practice in our setup with a view to improving biomechanical strength. The position achieved, calcar gap, nail diameter or length, blade length, Cleveland zone for the blade, and tip apex distance (TAD) were measured pre-operatively and endorsed in the data log. The reduction was categorized as “positive” if, on the AP view, the head-neck fragment was superomedial to the shaft fragment, and on the lateral view, the anterior cortex head-neck fragment was lying one cortical thickness above the shaft fragment (Figure 1).

**Figure 2:**
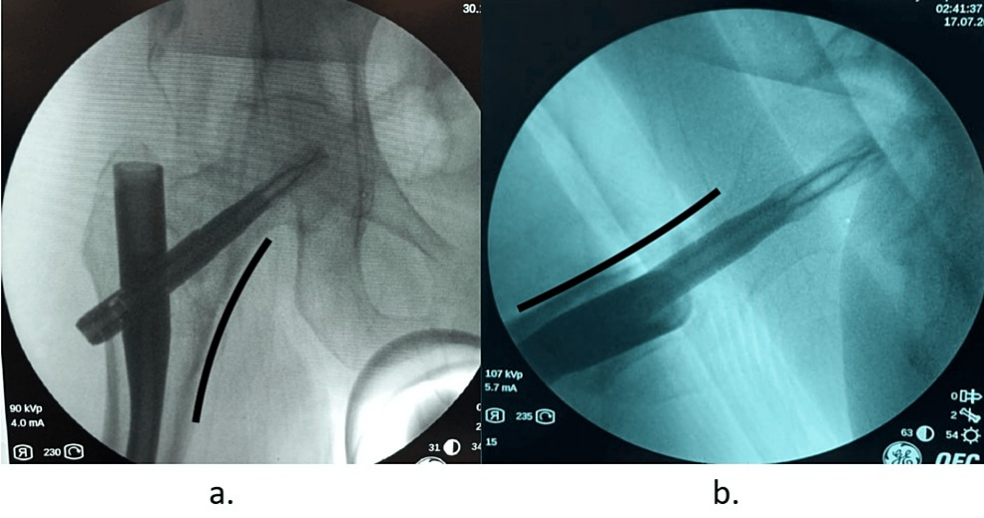
Neutral support in anteroposterior (a) and lateral (b) views The head-neck fragment is aligned with shaft fragment in anteroposterior (a) and lateral views (b).

The reduction was considered “neutral/anatomic” if, on AP and lateral view, the head-neck fragment is aligned with the shaft fragment (Figure 2).

**Figure 3:**
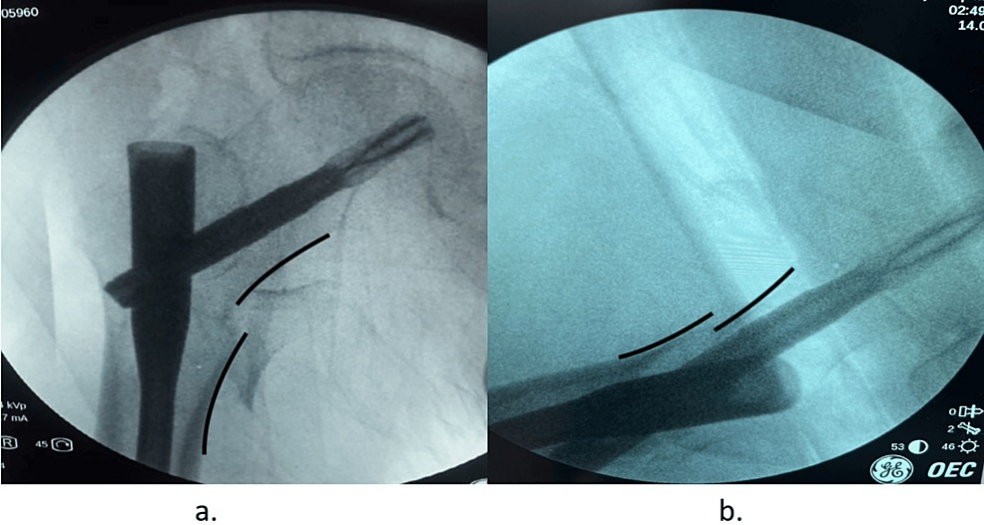
Negative contact in anteroposterior (a) and lateral (b) views of image intensifier (a) Anteroposterior view: the medial wall of the head-neck fragment is laterally displaced into the shaft fragment; (b) lateral view: showing step off more than one cortical thickness.

The reduction was categorized as “negative” if the medial wall of the head-neck fragment was laterally displaced into the shaft fragment on AP view and lateral view showed step-off or posterior sag of more than one cortex thickness (Figure 3).

The reduction was categorized as per Baumgartner’s criteria [16] and Chang’s criteria (Table 1).

**Table 1:** Chang's reduction quality criteria

Alignment	
Anteroposterior view: normal or slight valgus neck-shaft angle	1
Lateral view: less than 20° of angulation	1
Displacement	
Anteroposterior view: neutral or positive medial cortical support	1
Lateral view: smooth anterior cortical contact	1
Reduction quality	
Excellent	4
Acceptable	3 or 2
Poor	1 or 0

A protocol was devised to obtain AP radiographs of the operated and contralateral limbs postoperatively before discharge with 15° internally rotated. Isometric quadriceps, leg hanging bedside, and ankle pumps were instituted on the first post-operative day. Patients' discharge was individually tailored, and touch-down weight bearing was started as tolerated by the patient.

Patients were followed up for radiological calculation of parameters at three months and six months. Radiographs were obtained to measure neck length and neck-shaft angle in a true anteroposterior view of the hip. The construct was considered to have mechanical complications if there was gross cut-in, cut-out, cut-through, or implant breakage. The construct was also considered to have potential mechanical complications if the neck shaft angle loss was greater than 10° and the neck length loss was greater than 10 mm at six months. The functional score was calculated at approximately six months by the Hip Harris score. All radiological calculations were done by researchers not involved in operative technique per-operatively and kept blind to the operative data log (Areej Fatima). A digital console in the clinic connected to the radiology suite was used for calculations utilizing the WEASIS medical viewer™, version 3.6.2.

Data analysis was done using the Statistical Package for Social Sciences (SPSS) for Mac (released in 2016; IBM SPSS Statistics for Mac, version 24.0; IBM Corp., Armonk, NY). Mean and standard deviation for quantitative variables and frequency/percentages were computed for qualitative variables. Univariate analysis was done for demographics and variables pertinent to patients: fracture type, reduction technique, reduction manoeuvres, reduction quality, Cleveland zone, tip apex distance, and calcar gap. Neck shaft angle loss, neck length loss, tip apex distance, and Hip Harris score were compared utilizing an independent sample T-test. Cross tabulation and analysis were performed between Baumgartner’s grade, Chang’s grade, and mechanical complications utilizing the chi-square test. A p-value of less than or equivalent to 0.05 was considered significant.

## Results

A total of 202 patients were operated on from October 2021 until August 2023. Out of these, a six-month follow-up revealed documented mortality in 39 patients (19.3%). We lost follow-up with another 31 patients (15.3%), who were unreachable with the provided contact details on the first or second visit and were excluded from the study. For the remaining 132 patients, we were able to complete our clinical and radiological follow-up protocol.

The demographics and univariate analysis with reference to the developed mechanical complications are depicted in Table 2.

**Table 2:** Demographics and univariate analysis of patient characteristics (n=132) ^Quantitative variables; ^^qualitative variables; *utilizing independent sample T-test; ** Pearson’s chi-square test/Fischer exact test (P-value < 0.05 significant). The significant p-values were in bold. SD: standard deviation; CVA: cerebrovascular accident; AO: Arbeitsgemeinschaft für Osteosynthesefragen; NSA: neck Shaft angle; NL: neck length; HHS: hip Harris score.

S. No	Variable	Mean + SD^/n(%)^^ (n=132)	With complications (n=12)	Without complications (n=120)	P-value*
1.	Age	76.32 ± 7.98	81.0 ± 7.69	75.8 ± 7.89	0.795*
2.	Gender				
	Male	105 (79.5%)	9 (8.6%)	96 (91.4%)	0.682**
	Female	27 (20.5%)	3 (11.1%)	24 (88.9%)	
	Male/female	3.8:1	-	-	
3.	Comorbidities				
	Diabetes	54 (41%)	5 (9.3%)	49 (90.7%)	0.937**
	Hypertension	64 (48.4%)	6 (9.4%)	58 (90.6%)	
	Ischemic heart disease	7 (5.3%)	0	7 (100%)	
	CVA	7 (5.3%)	1 (14.3%)	6 (85.7%)	
4.	AO type				
	A2.2	67 (50.8%)	8 (11.9%)	59 (88.1%)	0.470**
	A2.3	65 (49.2%)	4 (6.2%)	61 (93.8%)	
5.	Reduction technique				
	Closed	58 (43.9%)	-	-	0.657**
	Hohmann	21 (16%)	-	-	
	Langenbeck	13 (9.8%)	-	-	
	Bone hook	26 (19.7%)	-	-	
	Periosteum elevator	14 (10.6)	-	-	
6.	Baumgartner’s				
	Good	72 (54.5%)	0	72 (100%)	<0.001**
	Satisfactory	48 (36.3%)	0	48 (100%)	
	Poor	12 (9.2%)	12 (100%)	0	
7.	Chang’s				
	Excellent	66 (50%)	0	66 (100%)	<0.001**
	Acceptable	60 (45.5%)	6 (10%)	54 (100%)	
	Poor	6 (4.5%)	6 (100%)	0	
8.	Cleveland zone				
	Centre-centre	54 (40.9%)	0	54 (100%)	<0.001**
	Inferior-centre	65 (49.2%)	6 (9.2%)	59 (90.8%)	
	Inferior-posterior	13 (9.9%)	6 (46.1%)	7 (53.9%)	
9.	Tip apex distance (mm)	24.56 ± 2.76	25.1 ± 2.75	24.5 ± 2.76	0.921*
10.	Calcar gap (mm)	5.16 ± 1.27	8.50 ± 0.52	4.83 ± 0.73	0.815*
11.	NSA loss (mm)	6.45 ± 2.09	11.5 ± 0.52	5.95 ± 1.41	<0.001*
12.	NL loss (mm)	6.50 ± 1.06	12.3 ± 0.75	5.55 ± 1.10	<0.001*
13.	HHS	69.27 ± 7.68	54.3 ± 13.13	69.7 ± 6.80	<0.001*

The mean age was 76.32 ± 7.98 years, with the minimum age being 65 and the maximum being 102 years of age. There were 105 male (79.5%) and 27 female (20.5%) patients, and the male-to-female ratio was 3.8:1. The known comorbidities were diabetes 54 (41%), hypertension 64 (48.4%), ischemic heart disease 7 (5.3%), and known patients with CVA 7 (5.3%). There were 67 (50.8%) A2 type and 65 (49.2%) A3 type fractures being operated upon. The two groups with and without mechanical complications were comparable and not significant statistically in terms of age (p = 0.795), gender (p = 0.682), comorbidity (p = 0.937), and fracture configuration type (p = 0.470). 

Closed reduction with the need for additional manoeuvres was required in 74 patients (56.1%), e.g., Homan’s, Cobb’s elevator, and bone hook, while 58 (43.9%) were successfully reduced by a simple closed traction table. The need for additional manouvres is not significant in terms of developing mechanical complications (p = 0.657). The mean tip apex distance achieved was 24.56 + 2.76 mm, and the calcar fracture gap residual after the achieved reduction was 5.16 ± 1.27. There was no statistical difference in terms of mechanical complications ascertained on univariate analysis, with p-values of 0.921 and 0.815, respectively. Cleveland zone centre-centre intent was executed but was achieved in 54 cases (40.9%), 65 cases (49.2%) ended up with inferior-centre, and 13 cases (9.9%) in inferior-posterior Cleveland zone. The value was statistically significant (p ≤ 0.001), and all complications were encountered in inferior-centre and inferior-posterior-positioned spiral blades.

**Figure 4:**
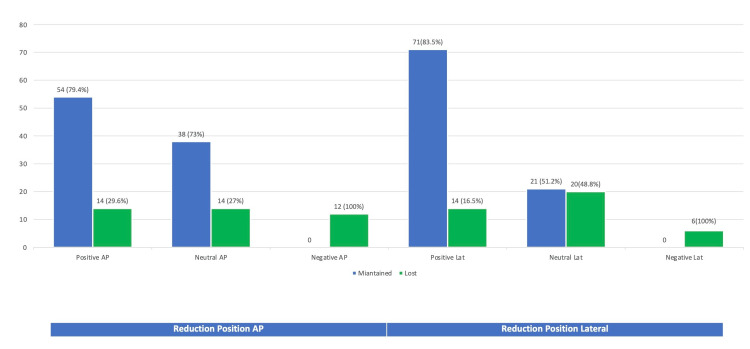
Primary loss of reduction in reference to per-operative position achieved The values are represented as the number of cases with a percentage, i.e., n (%).

The position achieved peroperatively was lost in the post-operative before discharge radiograph in 40/132 (30.3%). The details of maintained and lost reductions are depicted in Figure 4. Pertinent to mention is the loss of reduction of 14/52 (26.9%) in the neutral AP group and 20/41 (48.8%) in the neutral lateral group, as per the assessor kept blind to the per-operative position.

A total of 12 cases (9.09%) experienced mechanical complications. It included none of the gross complications, e.g., cut-in, cut-out, cut-through, or implant breakage at six months. All 12 cases were radiologically potentially constructed collapses as defined in the operational definition. The mean neck shaft angle loss was 6.45 ± 2.09, and the neck length loss was 6.5 ± 1.06, which was significantly lower in the mechanical complications group (p≤0.001) (Table 2).

**Figure 5:**
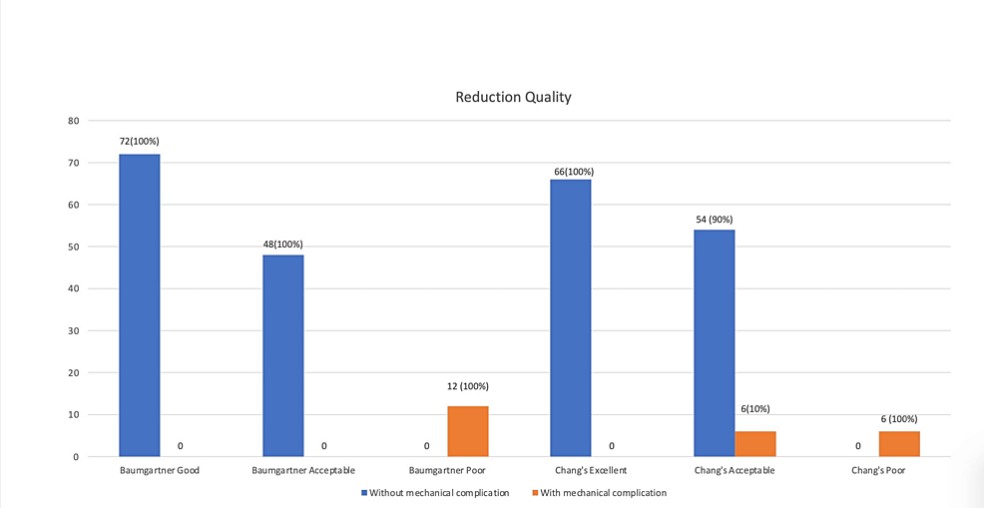
Quality of reduction and mechanical complications The values are represented as the number of cases with a percentage, i.e., n (%).

Figure 5 shows the frequencies of various grades of quality reduction as depicted by Baumgartner's and Chang’s criteria. Baumgartner’s good in 72 (54.5%), satisfactory in 48 (36.3%), and poor in 12 (9.2%) were graded. Chang's is excellent in 66 (50%), acceptable in 60 (45.5%), and poor in 6 (4.5%). Cross-tabulation reveals a significant association between the grading of Chang’s and Baumgartner’s poor groups for the development of mechanical complications (p<0.001).

The mean time to full weight bearing without support was 21 ± 1.22 weeks. The mean hip Harris score at six months was 69.27 ± 7.68.

## Discussion

In unstable pertrochanteric fractures, the quality of reduction is the key modifiable factor in the control of the operating surgeon to achieve optimal outcomes [17]. While functional outcomes are usually reported in terms of the scoring system, we chose neck shaft angle loss as the primary outcome measure, which can be represented in metrics to ascertain the impact of the reduction technique. Fixation in the varus is a known entity for post-fixation complications, i.e., cut-out, non-union, and limb length deficiencies [18,19].

Intramedullary proximal femoral nails are fixed-angle devices that are devised to provide superior biomechanical strength [20] and preserve neck-shaft angles in unstable fractures. They also facilitate the controlled telescoping of head-neck fragments, which results in secondary stability. Various criteria for reduction have been explained in the literature [21,22]. Baumgartner’s criteria have been in use in clinical practice, guiding the fracture fixation in the valgus in AP view and considering 4 mm fracture displacement. While theoretically guiding the restoration of the axis, it does not consider the secondary stability aspect in lieu of better fracture understanding in recent literature. Chang’s anteromedial reduction seems convincing because it not only incorporates angle maintenance in AP and lateral view but also caters to the contact position between the relevant anteromedial walls for secondary stability.

Our experience with Chang’s reduction has been pioneering in this part of the world. One of the obvious advantages is that it forced us to engage in additional manoeuvres in practice as part of our routine to achieve a stable position (74 patients, 56.1% in the current study). Those manoeuvres included the use of a bone hook, a Homan's spike, a periosteum elevator (Cobb's), or retractors. The implication of all complications in the Cleveland zone other than centre-centre suggests the need to attempt the guide wire in the centre-centre zone. The reduction position to be achieved perioperatively does not necessarily stay as such post-operatively due to various reasons [23,24], and it was obvious in our results as 40/132 (30.3%). The experience of the surgeon, the pixel resolution of the image intensifier, the ability of the blade to migrate, osteoporotic bone, and the fracture gap between the head, neck, and shaft fragment are all possible explanations. We were able to achieve positive anteromedial cortical support in 85/132 cases (64.3%), despite our intent to achieve it in all cases. Specifically, what appears to be cortex-to-cortex neutral alignment in lateral view can sag posteriorly, resulting in a posterior sag, which is a known factor for construct collapse [25]. In our study as well, lateral neutral alignment was lost to negative in 21/42 (48.8%).

Our 9.1% rate of mechanical complications (12 out of 132) is comparable to the reported rate of complications in the literature. Mao et al. reported 26 out of 127 patients (20.4%) being operated upon for unstable pertrochanetric fractures, engaging Chang’s criteria. The cut-out rate explained in various publications is 13% to 15% [26,27]. Studies have been published showing the impact of anteromedial support in controlling sliding and decreasing post-operative implant-related complications [28-30]. What appears to be a neutral per-operative may have a tendency to lose reduction and go negative upon initial weight bearing. In our series as well, the anteromedial support cases with an excellent Chang’s score had a better outcome as compared to the cases with neutral or negative support. Specifically, the neck shaft loss was significant if, in the lateral view, the contact was neutral or negative. The presence of no complications in Baumgartner’s satisfactory group but six complications in Chang’s acceptable group suggests the role of achieving all four parameters in Chang's criteria, which include positive support both in AP and lateral groups.

As far as the calcar gap is concerned, Baumgartner advocated a 4 mm gap as significant or developing complications. We have encountered difficulty in maintaining the calcar gap down to Baumgartner’s 4 mm, with our mean values being 5.16 ± 1.27 mm. Reducing the gap in both views might lead to varus fixation. We do not advocate that relying solely on the anteromedial buttress and ignoring the calcar gap can lead to good outcomes, but we think that NSA in slight valgus along with anteromedial support is crucial for stable construction along with placement of the implant in the appropriate Cleveland zone.

The strength of this study is that it is a prospectively conducted study, a single-arm study purposefully evaluating the radiological parameters. The calculation of radiological parameters was done unbiasedly by the assessor (AF), who was not part of the operating team and was kept blind to the per-operative data sheet. This study is the first of its kind to be done in this part of the world due to the recent introduction of access in clinics to DICOM images and handling consoles in clinics. The limitations are being a single-arm study with no control group available and reliability on radiographs for calculations of radiographic parameters. Furthermore, surgeon experience could have influenced the quality of the reduction, as it was performed by multiple surgeons in the current study. Future studies utilizing CT scans can better delineate post-operative radiological parameters and ascertain the impact of per-operative reduction.

## Conclusions

Anteromedial cortical support leads to less neck shaft angle loss, less neck length loss, and fewer implant-related mechanical complications at six months. The consideration of achieving a higher Chang’s grade drives surgeons to achieve optimal positions, utilizing additional manoeuvres for reduction. Our prospectively collected data suggests that anteromedial cortical support reduction and consideration of Chang’s criteria should be part of practice in theatres for optimal outcome achievement while fixing unstable pertrochanteric (31A2.2, A2.3) fractures.
